# Spatial clustering and determinants of home birth after at least one antenatal care visit in Ethiopia: Ethiopian demographic and health survey 2016 perspective

**DOI:** 10.1186/s12884-020-2793-6

**Published:** 2020-02-11

**Authors:** Atalay Goshu Muluneh, Yaregal Animut, Tadesse Awoke Ayele

**Affiliations:** 1 Department of Public Health Officer, College of Medicine and Health Sciences, University of Gondar, Gondar, Ethiopia; 2 Department of Epidemiology and Biostatistics, College of Medicine and Health Sciences, University of Gondar, Gondar, Ethiopia; 3 Gondar University Dabat research center, Gondar, Ethiopia

**Keywords:** Home delivery, Antenatal care, Spatial distribution, Ethiopia

## Abstract

**Background:**

All pregnancies are at risk and have to be attended by skilled professionals. In Ethiopia however nearly half (45.7%) of the women were giving birth at home after antenatal care (ANC) visits in which skilled professionals were not available. Therefore, the aim of this study was to assess spatial clustering and the determinant factors of home delivery after antenatal care visits in Ethiopia.

**Methods:**

A case control study was conducted on 2110 mothers who gave birth at home after ANC (cases), and 2510 mothers who gave birth at health institutions after attending ANC (controls), based on EDHS 2016 data. As per the recommendations of the DHS program, we weighed the data before analysis. ArcGIS 10.3 was used to show spatial pattern and SaTScan™ 9.4 to identify significant clusters. Stata 14 was used for data cleaning, weighing, and the analysis of the determinant factors. Bi variable and multi variable multilevel mixed effect logistic regression was fitted. Finally, the Log-likelihood ratio (LLR) and Relative risk with *p*-value of spatial scan statistics and AOR with 95% CI for significant determinant factors were reported.

**Results:**

Home delivery after ANC was spatially clustered in Ethiopia (Moran’s Index = 0.91, *p*-value< 0.01). Attending, 1–3 ANC visits (AOR = 1.41, 95%CI: 1.17–1.71), no information about birth preparedness plan (AOR = 2.21, 95%CI: 1.83–2.69), pregnancies wanted later (AOR = 1.55, 95%CI: 1.20, 2.06), not having health insurance (AOR = 2.16, 95% CI: 1.29, 3.62), Muslim (AOR = 1.57, 95% CI: 1.13, 2.19) and protestant (AOR = 1.72, 95%CI: 1.16, 2.42) religions were positively associated with home delivery; While being rich (AOR = 0.42, 95%CI: 0.32–0.54), middle wealth index (AOR = O.66, 95%CI: 0.51, 0.86), primary education (AOR = 0.45, 95%CI: 0.36–0.55), secondary education (AOR = 0.11, 95%CI: 0.07–0.16), above secondary education (AOR = 0.06, 95%CI: 0.03–0.11) were negatively associated.

**Conclusions:**

Home delivery after ANC follow ups was spatially clustered. Socio-demographic, health service and pregnancy related factors determined the prevalence of home delivery after antenatal care visits. Strengthening women’s education, ANC visit, giving more information about birth preparedness plan, and improving family wealth are vital to reduce home delivery after antenatal care visits.

## Background

All pregnancies are at risk and almost 15% pregnancies end up in life-threatening complications that need care from skilled personnel [1]. Globally, more than three hundred thousand mothers die each year due to complications of pregnancy and childbirth. Sub-Saharan Africa accounts for two thirds of the deaths, and Ethiopia is a bearer of a high burden of maternal mortality. Skilled birth attendance reduces complications during pregnancy and childbirth; it also delays maternal and child mortality as well as economic crises by creating healthy and productive communities [2–7]. All mothers attending ANC are expected to deliver at health institutions as increasing institutional delivery is one measure of the effectiveness of the ANC program. In Ethiopia however, nearly three-fourths of all pregnant women and half of the pregnant women who attend ANC deliver at home [8]. Home delivery has highly decreased globally, yet there still are great inequalities among countries and regions [7]. A study conducted in Nigeria showed that most clustered cases of non-skilled attendant deliveries were found in upper east and upper west regions [9]. Home delivery had spatial variations in Mozambique, with low-value (9%) clusters in the east and high value (41%) clusters in middle west [10]. Most home deliveries occur in the developing countries of Africa and Asia, including Ethiopia [2, 7]. Different factors predict home delivery in Ethiopia. Some studies were conducted on home delivery after ANC in Ethiopia and other parts of the world [11–14]. Socio-demographic, socio-economic, pregnancy and maternal health service related factors determine mothers’ choices of places of delivery in Ethiopia and other parts of the world [14–19].

Home delivery after ANC is a major public health problem in Ethiopia although limited studies have been conducted on the determinant factors of the practice. Therefore, the aim of this study was to explore spatial patterns and the determinants of home delivery after ANC in Ethiopia. The findings will help the policy makers and stakeholders to reduce home delivery after ANC, to minimize maternal and child mortality by designing locality specific interventions based on the identification of hotspot areas and factors that encourage women to give birth at home.

## Methods

### Study design

A case control study design was used to assess spatial clustering and the determinant factors of home delivery after antenatal care visits in Ethiopia. The Ethiopian Demographic and Health Survey (EDHS) data are collected at the national level every 5 years. A cross sectional study design is used to collect the survey data [20]**,** but for our objective, we used a secondary data analysis techniques to achieve our objective .

### Study area

Ethiopia is found in East Africa (3^o^-14^o^ N and 33^0^–48°E), and has 9 regional states (Afar, Amhara, Benishangul-Gumuz, Gambella, Harari, Oromia, Somali, Southern Nations, Nationalities and People’s (SNNP) and Tigray), and two administrative cities (Addis Ababa and Dire Dawa). Regions are divided into zones and each zone is divided into Woredas. Woredas are divided into the smallest administrative units called kebeles. Ethiopia is the second most populous country in Africa with high fertility rate of 4.6 children per woman. In Ethiopia, as in most African countries, women play the principal roles in the rearing of children and the management of family affairs. The Ethiopian Ministry of Health gives maternal health services, like delivery, antenatal and postnatal care services for free.

### Data source and measurements

The Demographic and Health Survey of Ethiopia (EDHS) collects data at the national level based on representative samples and key indicators, including maternal health conditions every 5 years. An Interviewer-administered questionnaire was used to collect data on women of reproductive age (15–49) years. The questionnaire included socio-demographic, socio economic pregnancy and maternal health service related variables on women’s health. A stratified two- stage cluster sampling was used to select 645 Enumeration Areas (EAs) (202 urban, 443 rural) with a probability proportional to EA size. A total of 15,683 women 7589 of whom delivered within 5 years before the survey were interviewed for ANC visit and place of birth. ANC visits were measured in numbers (0–20) and places of delivery were collected as respondents and relatives houses and health institutions (Table 1).

**Table 1:** List of variables used for analysis and their definition and measurement based on the EDHS 2016 report [20]

Variable name	Definition (Measurement)
Home delivery after ANC	Was defined as women who give birth at home after having at least one ANC visit within five years before the survey
Number of ANC visit	Was measured in number and mothers who had at least one ANC visit was considered as having ANC
Number of parity	The number of births given before the survey, including the most recent births among women who give birth within five years before the survey. Above 5 was considered as grand multiparous.
Age of respondent	Was age in year of the mothers who give birth within five years before the survey
Household wealth index	It was computed based on principal component analysis in the major DHS and the detail is available in the main DHS program
Covered by health insurance	Women were asked about did they have health insurance or not and reported as Yes/No in the EDHS program.
Informed about birth preparedness plan during their ANC visit	This was a Yes/ No question of about have you been informed about birth preparedness plan during ANC visit.
Last pregnancy wanted	The analysis was based on the last (Most recent) pregnancy. And Women were asked about the pregnancy was wanted or not.
Gestational age at 1st ANC visit	Gestational age at 1st ANC visit was the duration of the most recent pregnancy in months until the women had 1st ANC visit. ANC after 4 months was considered as late ANC visit.
Being exposed to media	Media exposure was calculated from the internet use, TV watching, radio listening, reading newspapers and those who score above the median were considered as having media exposure and the rest considered as having no media exposure.

**Fig. 1:**
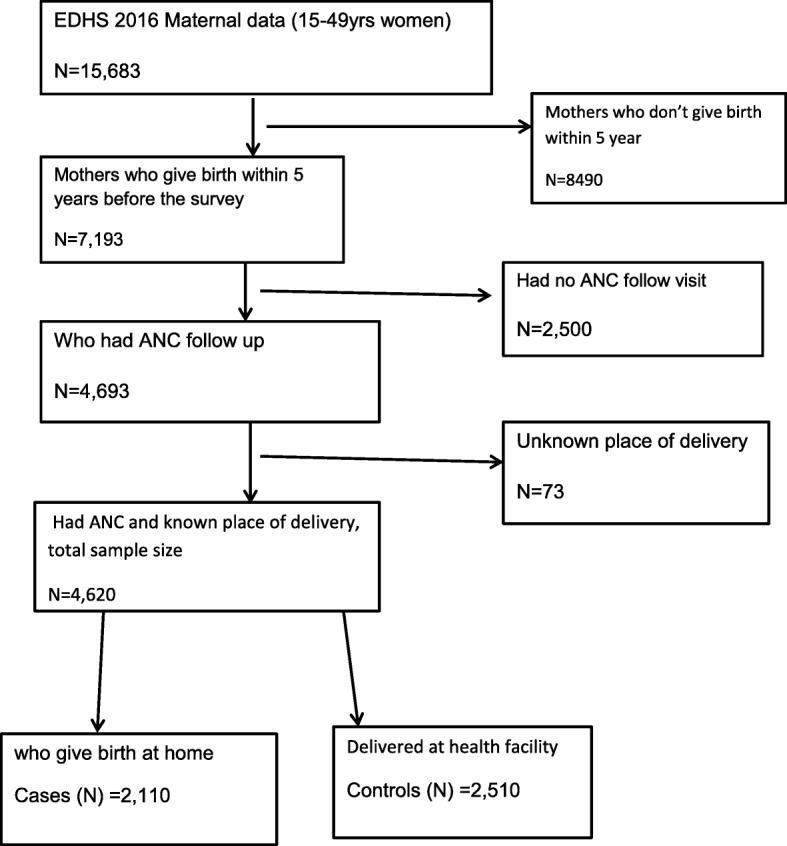
Flow diagram of how samples for our analysis were selected form EDHS 2016 maternal data

The geographic coordinate data was taken from each enumeration area [8]. A total of 2110 cases and 2510 controls were included in the analysis. Geographic coordinate data (latitude and longitude) were collected from selected enumeration areas. Coordinate data and other data set were accessed through online request from major DHS international (http://www.dhsprogram.com.) after registration as authorized users (Fig. 1). The shape file of the map of Ethiopia was accessed as an open source without restriction from Open Africa 2016 https://africaopendata.org/dataset/ethiopia-shapefiles.

### Data analysis

Since the sample was not proportionally allocated to different regions, and various non-response rates were detected, weighing the data was a must. We weighed data using women’s data weighing variable as recommended by the Ethiopian Demographic and Health Survey [8].

Geographic information system (ArcGIS) version10.3 and spatial scan statistics (SaTScanTM version 9.4) were used for spatial data analysis. Global and local level spatial autocorrelation analysis techniques were used to test the presence of spatial autocorrelation and spatial scan statistics were used for identifying significant and most likely clusters. Out of 643 clusters with geographic coordinates, we removed 21 and 15 clusters for having zero (invalid) GPS coordinates, and zero cases and controls, respectively. We analyzed 607 clusters with target population and valid GPS coordinates.

Global Moran’s index (Moran’s I) was used to test the presence of spatial autocorrelation at the national level. Global Moran’s I close to + 1 and − 1 represents spatial clustering and spatially dispersing respectively, while Global Moran’s I zero represents spatial randomness. A significant *p*-value< 0.05 and Global Moran’s I close to + 1 represent spatial clustering at the national level. Hot-spot analysis was conducted using Getis-Ord Gi* statistics to explore how spatial autocorrelation varied across the study areas. To determine the statistical significance of clustering, Gi Z-score was computed. A positive z-score > 1.96 with significant *p*-values represents hot-spot, while negative Z-score < − 1.96 with significant *p*-values represents Cold-spot [21]. Ordinary kriging interpolation was used to predict the unsampled data based on the sampled data creating a smooth surface to predict the burden of home delivery after ANC in the country [22].

Spatial scan statistics were conducted to identify significant and most likely clusters. SaTScanTM works with a moving window and requires fixing of the window size that moves across the study area. Since the outcome has Bernoulli distribution, the Bernoulli model was used by applying the Kuldorff method for purely spatial analysis. The model produces a log likelihood ratio (LLR), relative risk (RR) and *p*-value for each enumeration area with different radius. Areas with high LLR and significant *p*-value were considered as high risk areas compared to the outside of the window [23]. Finally, LLR, RR and *P*-values were reported for significant and most likely clusters.

For determinant factors, data cleaning and descriptive statistics were conducted first using STATA version 14. Home delivery had a significant clustering at the cluster level with an Intra-class correlation of 0.67 (0.62–0.72) and 0.44 (0.39, 0.5), respectively, for the null and, the final model. Multi-collinearity was checked using the Variance inflation Factor (VIF) and standard error. Types of place of residence had high VIF (> 10) and standard error of greater than 2. Finally, place of residence was excluded from the analysis due to multicolinearity. The data were correlated having intra-class correlation (ICC) = 0.67 (0.62, 0.72) and 0.44 (0.39, 0.50) for the null and final model, respectively which shows the data were significantly clustered. We fitted the mixed effect logistic regression model to get a better estimation of parameters. Bi-variable mixed effect logistic regression was conducted and variables with *p*-value < 0.25 were used in the multi-variable mixed effect logistic regression model. Finally, variables with significant level (*p* < 0.05) were reported with Adjusted Odds Ratio (AOR) and 95%CI as independent predictors of home delivery after ANC.

## Results

### Socio-demographic and economic characteristics of respondents

Of the 4620 participants, 2322 (49.73%) had no formal education; 1932 (41.38%) were Muslim. The mean age of the participants was 28.8 [SD = 6.6] years; most 4247 (90.94%) of the respondents were married, and nearly three-fourths, 3302 (70.72), were rural residents. Nearly a third, 1494 (31.99%) of the respondents were in the richest wealth index, while 3064 (65.61%) and 2510 (53.76%) of the women were not working and not exposed to the media respectively (Table 2).

**Table 2:** Socio-demographic characteristics of women who give home birth after at least one ANC visit in Ethiopia, EDHS 2016 perspective

Variable	Category	Weighed No of home delivery	Weighed No of institutional delivery	Total frequency (%)
Age of respondent				
	15–24	638	645	1283 (27.49)
	25–34	1077	1285	2362 (50.58)
	35–49	500	524	1024 (21.93)
Place of residence				
	Urban	271	1196	1367 (29.28)
	Rural	2044	1258	3302 (70.72)
Current marital status				
	Not married	166	257	423 (9.06)
	Married	2050	2197	4247 (90.94)
Respondents education				
	No formal education	1464	858	2322 (49.73)
	Primary	656	861	1517 (32.48)
	Secondary	72	411	483 (10.35)
	Higher	24	323	347 (7.43)
Respondents current occupation				
	Working	640	966	1606 (34.39)
	Not working	1575	1488	3064 (65.61)
Religion of respondents				
	Orthodox	659	1163	1822 (39.02)
	Muslim	1046	886	1932 (41.38)
	Protestant	474	373	847 (18.14)
	Other	36	32	68 (14.60)
Household wealth index of respondent				
	Poorest	707	282	989 (21.19)
	Poorer	450	301	751 (16.08)
	Middle	509	274	783 (16.78)
	Richer	336	315	651 (13.95)
	Richest	213	1281	1494 (31.99)
Exposed to media				
	No	1514	996	2510 (53.76)
	Yes	701	1458	2159 (46.24)

### Pregnancy and maternal health service related factors of women

Median gestational age at the 1st ANC visit was 5 [IQR = 2] months, ranging from 0 to 9 months, while the median frequency of ANC visits of mothers who had such visits and gave birth 5 years before the survey was 4 [IQR = 2] visits, ranging from 1 to 20 visits (Table 3).

**Table 3:** Pregnancy and maternal health service related factors of women who give home birth after at least one ANC visit in Ethiopia, EDHS 2016 perspective

Variable	Category	Weighted No of home delivery	Weighted No of institutional delivery	Total frequency (%)
Number of Parity				
	1–6	1848	2204	4052 (86.78)
	Above 6	368	249	617 (13.22)
Gestational age at 1st ANC visit				
	≤4 month	1066	766	1832 (39.23)
	After 4 months	1150	1688	2838 (60.77)
Number of ANC visit				
	1–3 visit	1239	896	2135 (45.71)
	≥4visit	977	1558	2535 (54.29)
Place (type of health facility) of ANC visit				
	Private health facility	56	206	262 (5.61)
	Public health facility	2084	2106	4190 (89.73)
	Other	75	142	217 (4.66)
Covered by health insurance				
	No	2158	2313	4471 (95.75)
	Yes	57	142	198 (4.25)
Informed about birth preparedness plan during ANC visit				
	No	1222	978	2200 (47.11)
	Yes	993	1476	2470 (52.89)
Pregnancy is wanted				
	Wanted then	1769	1942	3712 (79.49)
	Wanted later	333	384	717 (15.53)
	Wanted no more	112	129	241 (5.16)

The prevalence of home delivery after ANC has varied among regions in Ethiopia. The highest prevalence of home delivery after ANC found in Afar region was 70.55% (60.60, 78.87) and the lowest 2.41%, (0.89, 6.34) in Addis Ababa (Table 4)**.**

**Table 4:** Weighted prevalence of home birth after at least one ANC visit in Ethiopia across each region, EDHS 2016 perspectives

Region	Weighted frequencies		Weighted prevalence of home delivery after ANC (95% CI)
	Home delivery	Institutional delivery	
Tigray	181	440	29.16 (24.48, 34.33)
Afar	178	75	70.55 (60.60, 78.87)
Amhara	339	242	58.34 (52.18, 64.24)
Oromia	391	267	59.43 (52.41, 66.08)
Somali	226	120	65.28 (58.01, 71.91)
Benishangul –Gumuz	222	135	62.14 (54.26, 69.42)
SNNP^a^	382	257	59.79 (54.24, 65.09)
Gambela	128	149	46.26 (37.58, 55.18)
Harari	72	213	25.17 (18.58, 33.14)
Addis Ababa	9	354	2.41 (0.89, 6.34)
Dire Dawa	87	202	30.08 (21.97, 39.69)

^a^ South Nations, Nationalities and People region

### Spatial distribution of home delivery after antenatal care visit in Ethiopia

**Fig. 2:**
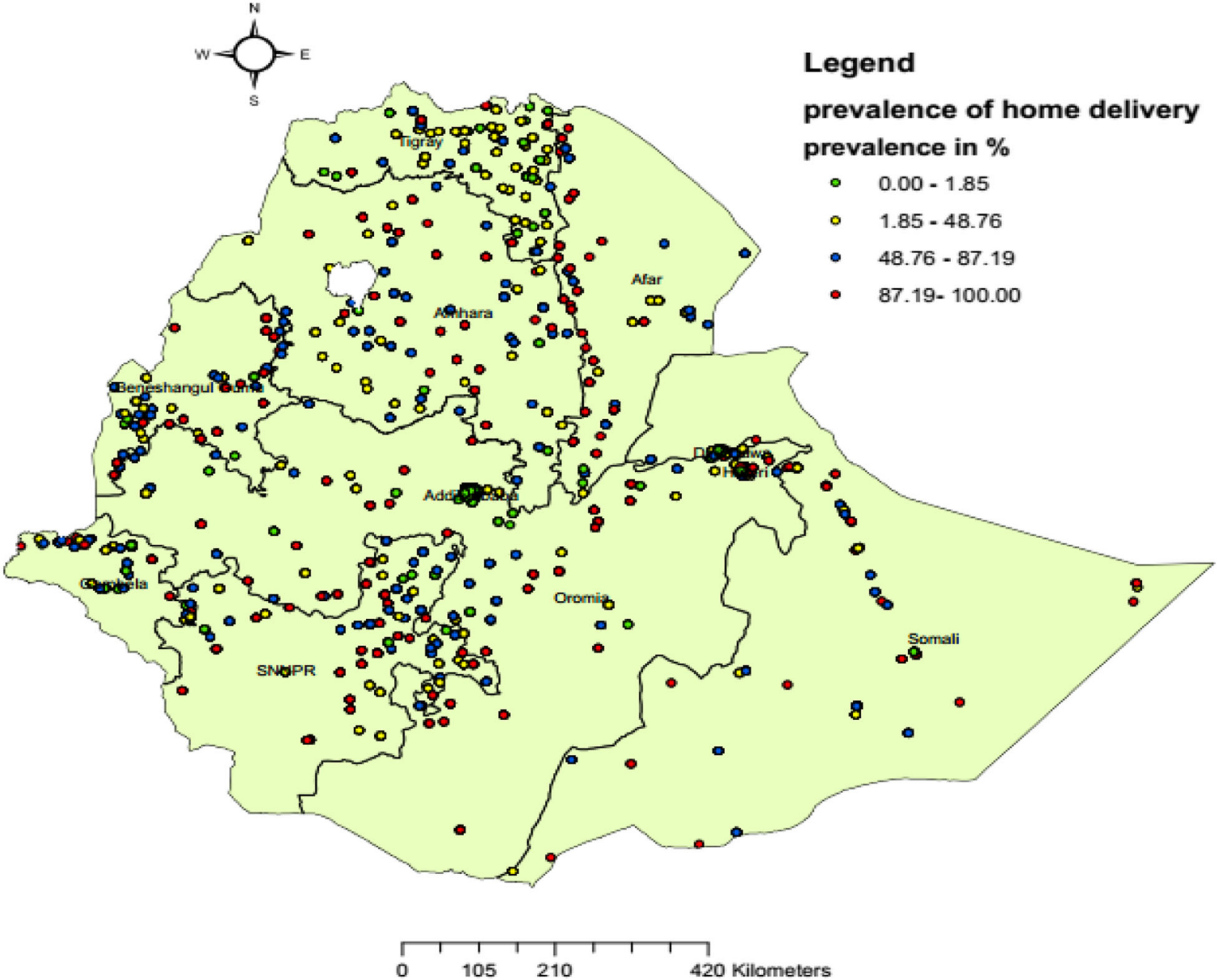
Spatial distribution of home birth after at least one ANC visit in Ethiopia, EDHS 2016 perspective. Each dot on the map represents one enumeration area

A total of 607 clusters were included in this analysis. Spatial distribution of home delivery after ANC in Ethiopia was mapped. Clusters with high prevalence of home delivery after ANC were found in north-west Somali, Benishangul-Gumuz (South, and East), SNNPR (East) and Amhara region (Central and East). Addis Ababa, Dire Dawa and Harari reported low prevalence of home delivery after ANC (Fig. 2).

**Fig. 3:**
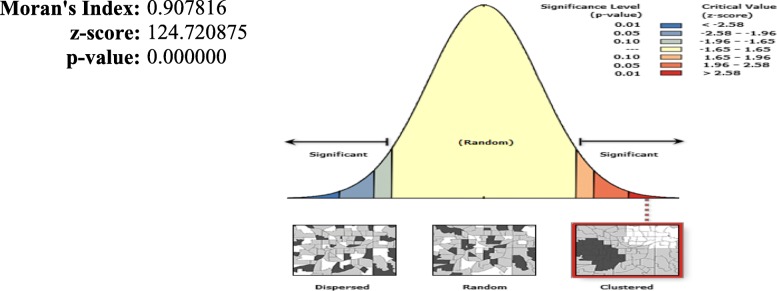
Spatial autocorrelation of home birth after at least one ANC visit in Ethiopia, EDHS 2016 perspective. Shape file of the map from ttps://africaopendata.org/dataset/ethiopia-shapefiles

The spatial distribution of home delivery after ANC visits was not completely random (Moran’s I = 0.91) in Ethiopia (Fig. 3). The presence of global spatial autocorrelation implies further spatial analysis techniques are important to identify significant cluster areas.

**Fig. 4:**
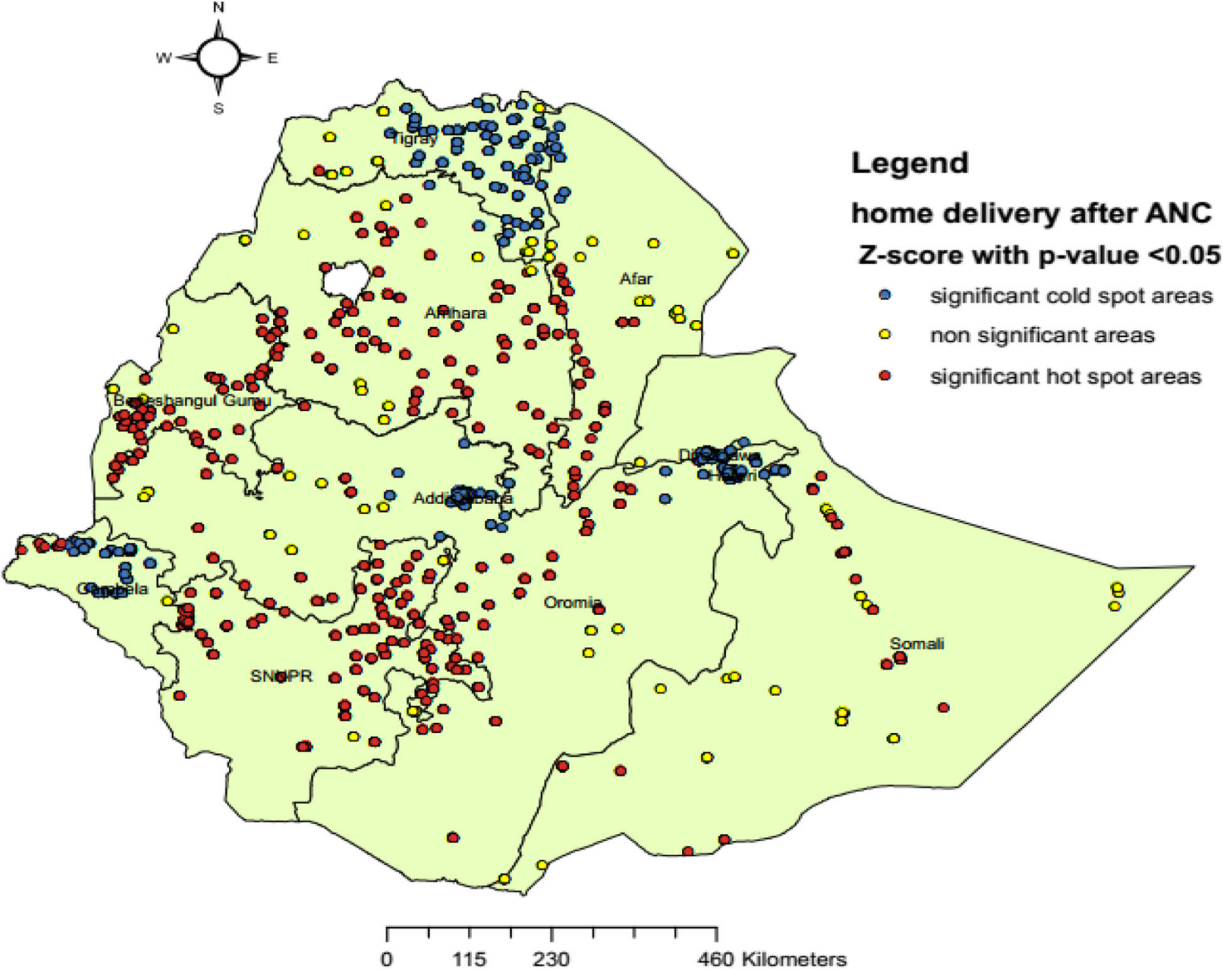
Hotspot analysis using getis ord G* statistics of home birth after at least one ANC visit in Ethiopia: EDHS 2016 perspective. A single dot on the map represents one enumeration Area. Shape file of the map from https://africaopendata.org/dataset/ethiopia-shapefiles

Hot-spot areas were found in Amhara (Central, and Southeast), Benishangul- Gumuz (Southwest, North, and Northeast), Oromia (Central), Afar (South), SNNP (Northwest, Central, South and Northeast), and Somali regions, while cold-spot areas were found in Tigray, Gambela (North, and Northeast), Addis Ababa, Harari and Dire Dawa (Fig. 4).

**Fig. 5:**
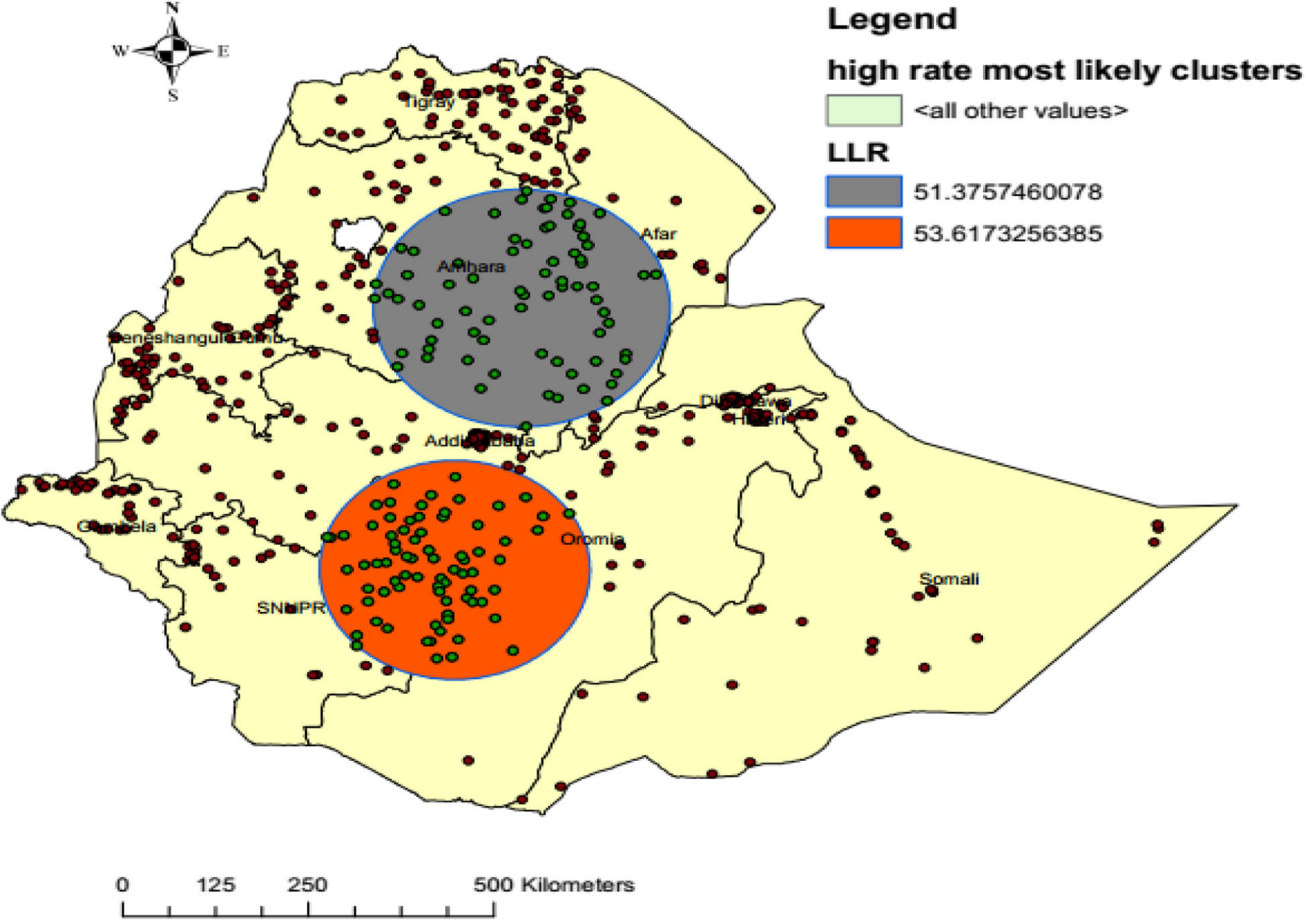
Most likely clusters of home birth after at least one ANC visit with 200 Kilometer radius in Ethiopia, EDHS 2016 perspective. A single dot on the map represents one enumeration area while the circles represent most likely clusters within 200 KM radius. LLR implies log likelihood ratio. Shape file of the map from https://africaopendata.org/dataset/ethiopia-shapefiles

Spatial scan statistics were done using SaTScan™ ^9.4^ to identify the most likely clusters and 137 most likely clusters in 200 km radius were identified. Of these, 76 and 61 enumeration areas were significant primary and secondary clusters, respectively. The primary most likely clusters were detected in central Oromia, central and northeast SNNP regions, while secondary clusters were found in Amhara, Afar and north Oromia (Fig. 5, Table 5).

**Table 5:** Significant and most likely clusters of home birth after at least one ANC visit in Ethiopia, EDHS 2016 perspective

Most likely cluster	Enumeration area (cluster) detected	Population	Cases	LLR	RR	Coordinate/radius	*P*-value
1st most likely clusters	308, 12, 391, 148, 216, 578, 365, 609, 408, 215, 576, 589, 420, 313, 347, 634, 468, 373, 26, 518, 405, 162, 388, 537, 445, 297, 20, 522,53, 600, 619, 360, 14, 41, 565, 272, 32, 633, 34, 271, 223, 180, 331,126, 232, 359, 316, 54, 141, 113, 438, 577, 182, 574, 434, 204, 562,502, 503, 505, 174, 306, 398, 213, 21, 142, 524, 217, 422, 227, 406,262, 450, 447, 486, 123, 86	657	418	53.62	1.52	(7.045432 N, 38.473023 E) / 180.88 km	< 0.001
2nd most likely clusters	617, 616, 354, 18, 460, 410, 345, 611, 267, 176, 10, 545, 310, 254, 496, 591, 637, 55, 368, 38, 478, 191, 547, 627, 510, 620, 571, 206, 189, 401, 572, 276, 295, 482, 102, 120, 389, 200, 229, 455, 423, 241, 201, 350, 332, 624, 484, 37, 344, 456, 66, 135, 24, 336, 249, 97	466	297	51.38	1.64	(10.939576 N, 39.281275 E) / 196.38 km	< 0.001

**Fig. 6:**
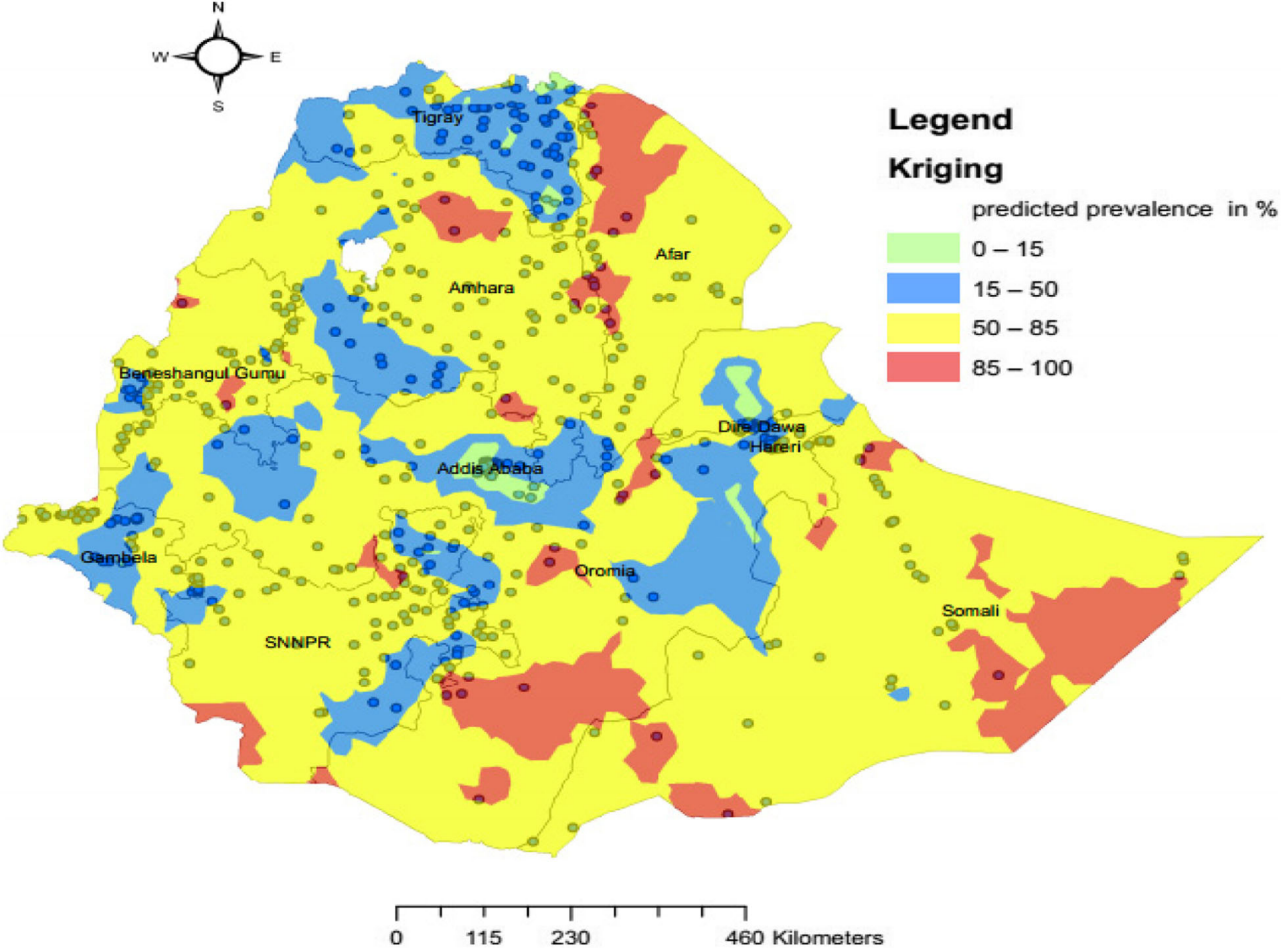
Ordinary kriging interpolation of home birth after at least one ANC visit in Ethiopia. Shape file of the map from https://africaopendata.org/dataset/ethiopia-shapefiles

Ordinary kriging interpolation was conducted to estimate the risk of home delivery after ANC in Ethiopia. Ordinary kriging works by creating a smooth surface to estimate the unsampled areas using available data (High-risk areas were found in Oromia (south and central), SNNP (south), Benishangul-Gumuz (southeast), Amhara (north, tip of south, and northeast) and Afar (northwest) were at high risk of home delivery (Fig. 6).

### Factors associated with home delivery after an ANC visit in Ethiopia

In the bi-variable Mixed effect logistic regression model, wealth index, respondent’s occupation, age, religion, pregnancy interest, place of ANC visit, number of ANC visits, media exposure, being informed about birth preparedness plans, parity, mothers education, marital status and having health insurance were significant at *p*-value< 0.25 and fitted the multi-variable analysis.

In the multi-variable mixed effect logistic regression model, we found that the number of ANC visits, religion, being informed about birth preparedness plans, mothers education, wealth index and being covered by health insurance were significantly associated with home delivery after ANC in Ethiopia.

Factors which indicated more likelihood of home delivery were, 1–3 ANC visits (AOR = 1.41, 95%CI: 1.17–1.71), lack of information on birth preparedness plans (AOR = 2.21, 95%CI: 1.83–2.69), absence of health insurance coverage (AOR = 2.16, 95%CI:1.29–3.62), affiliations with Islam (AOR = 1.57, 95%CI:1.13–2,19) and protestant church (AOR = 1.72, 95%CI:1.10–2.53). On the other hand, being rich (AOR = 0.42, 95%CI: 0.32–0.54) and middle wealth index (AOR = 0.66, 95%CI: 0.51–0.86), primary (AOR = 0.45, 95%CI: 0.36–0.55), secondary (AOR = 0.11, 95%CI: 0.07–0.16) and above secondary education (AOR = 0.06, 95%CI: 0.03–0.11) indicated less likelihood of delivery at home (Table 6).

**Table 6:** Mixed effect logistic regression analysis of home birth after at least one ANC visit in Ethiopia; EDHS 2016 perspective

Variable	Category	Home delivery	Institutional delivery	COR (95%CI)	AOR (95% CI)
Age of respondent					
	15–24	638	645	1	1
	25–34	1077	1285	1.06 (0.86, 1.30)	1.07 (0.86, 1.32)
	35–49	500	524	1.36 (1.05, 1.75)	1.18 (0.87, 1.86)
Number of parity of respondents					
	1–6	1848	2204	1	1
	Above 6	368	249	1.50 (1.17, 1.93)	1.26 (0.92, 1.72)
Number of ANC visit					
	≥4visit	977	1558	1	1
	1–3 visit	1239	896	1.59 (1.32, 1.91)	1.41 (1.17, 1.71) *
Respondents level of education					
	No education	1464	858	1	1
	Primary	656	861	0.46 (0.38, 0.56)	0.45 (0.36, 0.55) **
	Secondary	72	411	0.12 (0.08, 0.17)	0.11 (0.08, 0.18) **
	Higher	24	323	0.05 (0.03, 0.09)	0.06 (0.03, 0.11) **
Respondents current occupation					
	Not working	1575	1488	1	1
	Working	640	966	0.78 (0.64, 0.95)	0.86 (0.70, 1.06)
Religion of respondents					
	Orthodox	659	1163	1	1
	Muslim	1046	886	2.02 (1.42, 1.88)	1.57 (1.13, 2.19) *
	Protestant	474	373	1.48 (0.98, 2.23)	1.72 (1.16, 2.53) *
	Other	36	32	0.96 (0.40, 2.25)	1.03 (0.43, 2.42)
House hold wealth index of respondents					
	Poor	1155	574	1	1
	Middle	427	313	0.60 (0.46, 0.77)	0.66 (0.51, 0.86) **
	Rich	633	1567	0.35 (0.27, 0.45)	0.42 (0.32, 0.54) **
Being exposed to media					
	No	1514	996	1.65 (1.34, 2.04)	1.18 (0.95, 1.48)
	Yes	701	1458	1	1
Type of health facility for ANC visit					
	Private facilities	56	206	0.46 (0.27,0.77)	0.60 (0.35, 1.03)
	Public facilities	2084	2106	1	1
	Other^1^**	75	142	0.72 (0.45, 1.17)	0.71 (0.43, 1.15)
Covered by health insurance					
	No	2158	2313	2.13 (1.39, 3.84)	2.16 (1.29, 3.62) *
	Yes	57	142	1	1
Informed about birth preparedness plan during their ANC visit					
	No	1222	978	2.34 (1.93, 2.83)	2.21 (1.83, 2.69)*
	Yes	993	1476	1	1
Last pregnancy is wanted					
	Wanted then	1769	1942	1	1
	Wanted later	333	384	1.38 (1.08, 1.77)	1.55 (1.20, 2.01) *
	Wanted no more	112	129	1.22 (0.82, 1.82)	1.05 (0.70, 1.57)
Gestational age at 1st ANC visit					
	≤4 month	1066	766	Not sig(*p* = 0.28)	
	After 4 months	1150	1688		
Current marital status					
	Not married	166	257	not sig (*p* = 0.29)	
	Married	2050	2197		
ICC for the null model			o.67 (0.62, 0.72)		
ICC for the final model			0.44 (0.39,0.50)		

^1^** place of ANC visit represents home and unspecified other places; *positively associated; **negatively associated

*COR* Crude Odds Ration, *AOR* Adjusted Odds Ratio, *CI* confidence interval.

1 = represents a reference category.

## Discussion

This study was conducted to assess spatial clustering and determinant factors of home birth after at least one ANC visit in Ethiopia. We found that home delivery after ANC in Ethiopia was clustered and affected by different socio-demographic, pregnancy and maternal health service related factors.

The spatial distribution of home delivery after ANC in Ethiopia was clustered at national and regional levels. Home delivery after ANC had spatial dependency (Moran’s I: 0.91), and significant clusters were found in different regions of the country.

Amhara, Oromia, SNNP, Benishangul-Gumuz and some parts of Afar were areas at high risk for home delivery after ANC, while Addis Ababa, Dire Dawa, Harari, Tigray and Gambela were low-risk regions. The possible justification for this may be that regions like Addis Ababa, Tigray, Dire Dawa and Harari were urban areas where most of the mothers had better education and access to health facilities compared to the other regions [24]. For Gambela region, different activities that encourage women to deliver at health facilities may help them to deliver at institutions. Unpublished reports showed that Gambela region strengthened effort for institutional delivery as of 2006 and achieved a very dramatic increase of 167% from 2006 to 2010 [25]. The reason for this might be that regions like Gambella had a low institutional delivery rate earlier and that the Ethiopian Federal Ministry of Health and other stakeholders, like WHO, gave the issue a high emphasis. Additionally, the fact that the region had to host many refugees helped it to attract more attention [26, 27].

Household wealth index was negatively associated with home delivery after ANC. Mothers who had rich and middle income had 58, and 34% less chances of delivering at home after ANC visits respectively as compared to the poor. This finding is supported by those of other studies conducted in Ethiopia [11], rural Mozambique [10], Kenya [28] and Afghanistan mortality survey [29], where wealth index was independently associated with non-institutional delivery. Women who had primary, secondary and above secondary education were 55, 89 and 94% less likely to deliver at home respectively as compared to mothers who had no formal education. These findings are supported by the results of studies conducted in Afar region [30], north-west Ethiopia [11, 13] and Afghanistan [29].

Inadequate ANC visits increased the chances of home delivery after ANC. Mothers who had 1–3 ANC were 1.41 times more likely to deliver at home compared to mothers who had more than 4 visits. This finding is supported by a systematic review conducted in 2014 [18] and other studies in Ethiopia [16], Nigeria [31] and Afghanistan [29]. A possible justification may be that mothers who had adequate ANC visits (> 4) may have information about the benefits of institutional delivery, positive attitude towards maternal health care services, including place of delivery, the risk of pregnancy and childbirth.

Mothers who were not told about birth preparedness plans during their ANC visits had more than double chances of delivering at home after ANC visits in Ethiopia. Studies conducted in Ethiopia [32] and Bengal [33] show that birth preparedness and complication readiness were independent predictors of choices of places of delivery.

Mothers who were not covered by the health insurance were two times (AOR = 2.16, 95%CI: 1.29–3.62) more likely to deliver at home. This finding is supported by those results of studies conducted in Kenya [28] and Nigeria [34]. Pregnancies that were not planned at the times were more likely to be delivered at home. This finding is supported by the results of studies conducted in Ethiopia, where unplanned pregnancies were more than 3 times more likely to be delivered at home [11].

### Implications of the study

This study identified that increasing the frequency of ANC visits, encouraging women to have better education, and the identifying significant hotspot clusters were essential preconditions for interventions. As unexpectedly high numbers of mothers give birth at home after ANC, encouraging institutional delivery is very important.

### Strength of the study

That it was based on nationally representative data, might have helped to make a better prediction of parameters.

### Limitation of the study

Although the study had its own strengths, we faced limitations in that we used secondary data; behavioral factors like the attitude of women on home delivery, quality of services and traditions of birth were difficult to assess. The distortion of location data (1-2KM for urban and 1-5KM for rural) for security purpose might have affected knowledge of the specific location of cases.

## Conclusions

Home delivery after ANC was spatially clustered. Significant and most likely clusters were found in Amhara, Oromia, Afar and southern Nation’s Nationalities and Peoples regions.

Having formal education and being rich reduce the chances of home delivery after ANC. On the other hand, having less number of ANC visits, not being covered by health insurance, lack of information about birth preparedness plans during ANC visits, pregnancies wanted later encouraged women to give birth at home after antenatal care booking in Ethiopia.

## Data Availability

The data used for preparation of this manuscript are available from http://www.dhsprogram.com and anyone can access through online request as authorized user. The shape files of the maps Ethiopia were freely accessible at https://africaopendata.org/dataset/ethiopia-shapefiles**.** The authors prepared the data that was used for preparation of this manuscript can be shared if required.

## Abbreviations

ANC: Antenatal Care
AOR: Adjusted Odds Ratio
EA: Enumeration Area
EDHS: Ethiopian Demographic and Health Survey
LLR: Log Likelihood Ratio
RR: Relative Risk

## References

[1] World Health organization, Department of Maternal N, Child and adolescent health (2017). Family, Women’s and Children’s Health Integrated Management Of Pregnancy And Childbirth.

[2] Global health obseervatory. Skilled birth attendant situations and trends. 2018. https://www.who.int/gho/maternal_health/skilled_care/skilled_birth_attendance_text/en/.

[3] WHO (2018). WHO recommendations Intrapartum care for a positive childbirth experience. SWIZERLAND Department of Maternal, Newborn, Child and Adolescent Health.

[4] WHO (2016). maternal mortality fact sheet 2016. WHO media center.

[5] Kassebaum NJ, Barber RM, Bhutta ZA, Dandona L, Gething PW, Hay SI (2016). Global, regional, and national levels of maternal mortality, 1990–2015: a systematic analysis for the global burden of disease study 2015. Lancet.

[6] UNFPA (2013). MATERNAL HEALTH IN AFRICA Adis Ababa: UNFPA.

[7] Montagu D, Sudhinaraset M, Diamond-Smith N, Campbell O, Gabrysch S, Freedman L (2017). Where women go to deliver: understanding the changing landscape of childbirth in Africa and Asia. Health Policy Plan.

[8] Central Statistical Agency (CSA) [Ethiopia] and ICF. Ethiopia Demographic and Health Survey 2016. Addis Ababa: CSA and ICF; 2016.

[9] Gayawan E (2014). Spatial analysis of choice of place of delivery in Nigeria. Sex Reprod Healthc.

[10] Agadjanian V, Yao J, Hayford SR (2016). Place, Time and Experience: Barriers to Universalization Of Institutional Child Delivery in Rural Mozambique. Int Perspect Sex Reprod Health.

[11] Kasaye HK, Endale ZM, Gudayu TW, Desta MS (2017). Home delivery among antenatal care booked women in their last pregnancy and associated factors: community-based cross sectional study in Debremarkos town, north West Ethiopia, January 2016. BMC Pregnancy Childbirth.

[12] Choe S-A, Kim J, Kim S, Park Y, Kullaya SM (2015). Kim C-y. do antenatal care visits always contribute to facility-based delivery in Tanzania? A study of repeated cross-sectional data. Health Policy Plan.

[13] Desalegn E, Mekonnen A, Abeje G (2014). Place of delivery after antenatal care: the case of Fogera district, Amhara region, north west, Ethiopia; 2013. J Gynecol Obstet.

[14] Araya M, Y A KA (2016). Spatial Distribution and Associated Factors of Women’s Home Delivery after Antenatal Care Visit in Lay Gayint District, Northwest Ethiopia.

[15] Kananura RM, Tetui M, Mutebi A, Bua JN, Waiswa P, Kiwanuka SN (2016). The neonatal mortality and its determinants in rural communities of eastern Uganda. Reprod Health.

[16] Wilunda C, Quaglio G, Putoto G, Takahashi R, Calia F, Abebe D (2015). Determinants of utilisation of antenatal care and skilled birth attendant at delivery in south west Shoa zone, Ethiopia: a cross sectional study. Reprod Health.

[17] Tarekegn SM, Lieberman LS, Giedraitis V (2014). Determinants of maternal health service utilization in Ethiopia: analysis of the 2011 Ethiopian demographic and health survey. BMC Pregnancy Childbirth.

[18] Berhan Y, Berhan A (2014). Antenatal care as a means of increasing birth in the health facility and reducing maternal mortality: a systematic review. Ethiop J Health Sci.

[19] Abebe F, Berhane Y, Girma B (2012). Factors associated with home delivery in Bahirdar, Ethiopia: a case control study. BMC Res Notes.

[20] Central Statistical Agency (CSA) [Ethiopia] and ICF (2016). Ethiopia Demographic and Health Survey 2016.

[21] Pfeiffer D, Robinson TP, Stevenson M, Stevens KB, Rogers DJ, Clements AC (2008). Spatial analysis in epidemiology: Oxford University press Oxford.

[22] Lam NS-N (1983). Spatial interpolation methods: a review. Am Cartographer.

[23] Kulldorff M (1997). A spatial scan statistic. Commun Stat Theory Methods.

[24] Kebede A (2017). Ethiopia service availability and readiness assessment 2016 Addis Ababa.

[25] Bwalya’ S, Alebachew S. Analyzing Performance and Regional Disparities in Health Outcomes in Ethiopi*a*. *ethiopia*; 2012. Report No.: 2

[26] UNDP ETHIOPIA (2012). Analyzing performance and regional disparities in health outcomes in Ethiopia.

[27] World Health Organization Africa. WHO Ethiopia supports Gambella region to stay a step ahead of impending health threats in the rainy season. https://www.afro.who.int/news/who-ethiopia-supports-gambella-region-stay-step-ahead-impending-health-threats-rainy-season.

[28] Kitui J, Lewis S, Davey G (2013). Factors influencing place of delivery for women in Kenya: an analysis of the Kenya demographic and health survey, 2008/2009. BMC Pregnancy Childbirth.

[29] Azimi MD, Najafizada SAM, Khaing IK, Hamajima N (2015). Factors influencing non-institutional deliveries in Afghanistan: secondary analysis of the Afghanistan mortality survey 2010. Nagoya J Med Sci.

[30] Abdella M, Abraha A, Gebre A, Surender RP (2017). Magnitude and associated factors for home delivery among women who gave birth in last 12 months in Ayssaita, Afar, Ethiopia-2016. Community Based Cross Sectional Study Glob J Fertil Res.

[31] Dahiru T, Oche OM. Determinants of antenatal care, institutional delivery and postnatal care services utilization in Nigeria. Pan African Med J. 2015;21(1). 10.11604/pamj.2015.21.321.6527. 10.11604/pamj.2015.21.321.6527 PMC4633744 26587168

[32] Tura G, Afework MF, Yalew AW (2014). The effect of birth preparedness and complication readiness on skilled care use: a prospective follow-up study in Southwest Ethiopia. Reprod Health.

[33] A D GA. Status of birth preparedness and complication readiness among recently delivered women: a community based study in a slum of Kolkata, West Bengal. Int J Community Med Public Health. 2017;4(9).

[34] Okusanya BO, Roberts AA, Akinsola OJ, Oye-Adeniran BA (2016). Birth plans and health insurance enrolment of pregnant women: a cross-sectional survey at two secondary health facilities in Lagos, Nigeria. J Matern Fetal Neonatal Med.

